# The impact of a lay counselor led collaborative care intervention for common mental disorders in public and private primary care: A qualitative evaluation nested in the MANAS trial in Goa, India^☆^

**DOI:** 10.1016/j.socscimed.2013.04.002

**Published:** 2013-07

**Authors:** Sachin Shinde, Gracy Andrew, Omer Bangash, Alex Cohen, Betty Kirkwood, Vikram Patel

**Affiliations:** a Sangath, Goa, India; b London School of Hygiene & Tropical Medicine, London, UK; c Centre for Global Mental Health, London School of Hygiene & Tropical Medicine, London, UK; d Faculty of Epidemiology and Population Health, London School of Hygiene &Tropical Medicine, London, UK; e Center for Mental Health, Public Health Foundation of India, India

**Keywords:** India, Depression, Anxiety, Qualitative, Lay health workers, Randomized controlled trial, Primary care

## Abstract

The MANAS trial evaluated the effectiveness of a lay counselor led collaborative stepped care intervention for Common Mental Disorders (CMD) in public and private sector primary care settings in Goa, India. This paper describes the qualitative findings of the experience of the intervention and its impact on health and psychosocial outcomes. Twenty four primary care facilities (12 public and private each) were randomized to provide either collaborative stepped care (CSC) or enhanced usual care (EUC) to adults who screen positive for CMDs. Participants were sampled purposively based on two criteria: gender and, in the CSC arm, adherence with the intervention. The qualitative study component involved two semi-structured interviews with participants of both arms (*N* = 115); the first interview within 2 months of recruitment and the second 6–8 months after recruitment. Data were collected between September 2007 and November 2009. More participants in the CSC than EUC arm reported relief from symptoms and an improvement in social functioning and positive impact on work and activities of daily life. The CSC participants attributed their improvement both to medication received from the doctors and the strategies suggested by the lay Health Counselors (HC). However, two key differences were observed in the results for the two types of facilities. First, the CSC participants in the public sector clinics were more likely to consider the HCs to be an important component of providing care who served as a link between patient and the doctor, provided them skills on stress management and helped in adherence to medication. Second, in the private sector, doctors performed roles similar to those of the HCs and participants in both arms placed much faith in the doctor who acted as a confidante and was perceived to understand the participant's health and context intimately. Lay counselors working in a CSC model have a positive effect on symptomatic relief, social functioning and satisfaction with care in patients with CMD attending primary care clinics although the impact, compared with usual care, is greater in the public sector.

## Highlights

• Qualitative evaluation of a lay counselor primary mental health care intervention.
• The intervention led to improved patients' health and social outcomes.
• Improvements were attributed to both counselling and medicines.
• Differences were greater in the public compared with the private facilities.

## Introduction

Depressive or anxiety disorders ('common mental disorders' or CMD) are the leading psychiatric causes of the global burden of disease and the vast majority of patients with these disorders present in primary health care (Marcus, Yasamy, Ommeren, Chisholm, & Saxena, 2012). In spite of robust evidence that supports the effectiveness of brief psychological treatments and antidepressants for these disorders (World Health Organization, 2001); a large treatment gap is observed in low resource settings due to various barriers faced for mental health care in primary care settings (Kohn, Saxena, Levav, & Saraceno, 2004). Notable among these barriers is the lack of skilled and affordable human resources in such settings to deliver the psychosocial interventions and support adherence with medication. The MANAS trial (MANashanti Sudhar Shodh, which means “project to promote mental health” in the Konkani language) was aimed at evaluating the effectiveness of a collaborative stepped care (CSC) intervention, against the enhanced usual care (EUC), for CMD that was led by lay counselors in government-run primary health centers (PHCs) and private general practitioner (GP) settings in Goa, India. Full details of the intervention and the evaluation have been published elsewhere (Chatterjee, Chowdhary, Pednekar, & Cohen, 2008; Patel, Weiss, Chowdhary, & Naik, 2010; 2011). Box 1 provides the detailed overview of the MANAS trial. The major results of the quantitative outcome measures revealed that although the intervention was consistently associated with strong beneficial effects on the mental health and functional status of participants in the PHCs, there was little evidence of impact of the intervention on outcomes among participants attending GP facilities, principally because the comparison arm facilities did as well as the intervention facilities.

There is an increased recognition of the value of embedding qualitative research in RCTs as qualitative data can offer explanations of the processes and intervening factors that yielded quantitative outcomes (Hawe, Shiell, Riley, & Gold, 2004; Lewin, Glenton, & Oxman, 2009; Oakley, Strange, Bonell, Allen, & Stephenson, 2006) and in evaluating outcomes which are difficult to assess using quantitative tools (e.g., subjective experiences of the quality of care). Thus, the qualitative approach not only helps unpack the contextual factors or intervention characteristics that may have influenced the trial results but also increases the range of outcomes which might be evaluated in the trial (Bower, Gilbody, Richards, & Flecher, 2006; Glenton, Lewin, & Scheel, 2011). The major aim of the qualitative study described in this paper was to explore the experience of the addition of a lay counselor, and the role of key other members of the primary care team, to address CMDs in public and private primary care. A secondary aim was to explore and compare the impact of the intervention on health and social functioning between the two arms. The analyses were carried out separately for each of the two types of facilities in order to understand better the reasons for the differential quantitative findings. The analyses in this article focus on the experiences of participants; the experiences of providers have been published elsewhere (Pereira, Andrew, Pednekar, Kirkwood, & Patel, 2011) and the findings of the two analyses are triangulated in the discussion.

## Methodology

### Sample

The qualitative study sample was recruited after meeting the sample targets of quantitative trial, but following the same criteria as the main trial (Box 1) with the sole exception that only patients with moderate to severe CMD, i.e. a higher cut-off score on the General Health Questionnaire-12 were selected (Patel, Araya, Chowdhary, King, Kirkwood, 2008). Of those who met these criteria and were screened positive, purposive sampling was then carried out to meet two criteria: we over-sampled females to be consistent with the demographic characteristics of patients with CMD in primary care and, in CSC attendees; we sampled participants to reflect a diverse level of adherence with the intervention. In this way, in each EUC facility, 3 female and 1 male participant and in each CSC facility, 6 female and 2 male participants were purposively invited to participate. Table 1 reports the total numbers of participants recruited, their characteristics and follow-up rates. In summary, 69 CSC and 46 EUC participants took part in the study of whom 79% (*n* = 55) and 82.6% (*n* = 38) respectively completed the follow up interview.

### Data collection and analysis

Data were collected through two semi-structured interviews; the first interview was conducted about 2 months after recruitment while the second interview was conducted between 6 and 8 months after recruitment. Data for the PHC phase were collected between September 2007 and July 2008 and for the GP phase between December 2008 and November 2009. Interviews were mostly at the participants' home, with one exception (where the participant preferred PHC as the location of the interview). Most interviews were conducted in Konkani (113/115; the remainder in English) by a team of four trained field researchers. On an average, the first interview lasted for 45 min to an hour while the follow up interview took around 30 min. The first interview guide addressed the participant's experience of the illness before recruitment in the trial; changes in health and social well-being since recruitment; and the participant's experiences about the various treatment components (based on the allocation arm). In the follow up interview, the participant's health and social well being were reviewed once more and gaps in the information from the first interview were addressed.

All interviews were audio-taped; the recorded interviews were transcribed verbatim and local language interviews were translated into English. Subsequently, the memos (reflective notes about the interviews) and field notes written by the interviewers were attached to the main text of the interviews. We used thematic analysis (Aronson, 1994; Auerbach & Silverstein, 2003, Chap. 6 & 7; Braun & Clarke, 2006; Miles & Huberman, 1994, Chap. 4 & 5; Strauss, 1987, Chap. 1; Part II). In the first stage, two authors (SS & GA) read and familiarized themselves with the data. In the second stage, the two authors (SS & GA) selected 10 interviews and generated initial codes through coding parcels of data in a systematic fashion (Miles & Huberman, 1994, Chap. 4 & 5). Third, based on the coded data and the original research questions, the researchers defined and collated codes into potential themes, and finalized the code-book. The major identified themes were: role and experience of the HC, HA and primary care doctor; baseline experience of symptoms and social functioning; and change and attribution of change over time in these outcomes. In the fourth stage, the inter-rater reliability was tested by double coding 10 randomly selected interview scripts (Miles & Huberman, 1994, Chap. 4 & 5), i.e. four researchers (including two researchers who worked during the earlier stages) coded the same documents and discussed the agreements and disagreements. The inter-rater reliability i.e. total number of agreements divided by total number of comparisons was around 85%. Then these four researchers coded the entire data set. In the final stage, two researchers (SS & GA) tallied simple frequencies for major themes (Neuendorf, 2002, Chap. 3), selected and analyzed vivid and compelling examples of narrative extracts, and related these to the research questions. Data were coded in Atlas Ti software (version 4.2).

### Ethical considerations

Written informed consent was obtained from the participants on their first visit to the PHC and GP clinics. Respondents were given a choice regarding the location of interview and were assured about confidentiality and voluntary nature of participation that would have no impact on treatment provided to them. Verbal consent was taken again at the time of each of the two interviews. Ethical approval for the study was obtained from the Institutional Review Board of Sangath, India as well as of The London School of Hygiene and Tropical Medicine, UK.

## Results

### Sample characteristics

Altogether, we interviewed 115 participants (PHC-CSC: 31, PHC-EUC: 23; GP-CSC: 38, GP-EUC: 23) of whom 81% (44 from PHC; 49 from GP phase) completed the second interview. The average age was 48.2 years (Range 20–86; SD 13.8). There were 85 women (51 from CSC; 34 from EUC) and 30 men (18 from CSC; 12 from EUC). The average GHQ score was 8.8 (Range 8–12; SD 0.9). Half of women participants reported being a housewife (*n* = 42; 50%), while for male participants the most common occupation was daily wage labor (*n* = 22; 73.5%). There were no significant differences in terms of age, gender, education, occupation, and GHQ-12 scores of participants between CSC and EUC arm. Table 1 shows the age, gender, and GHQ score distribution in CSC and EUC arm for each facility type.

### Quality of care

There were three key human resources which the CSC arm participants encountered, viz. the HA (who carried out the screening), the doctor and the HC. The EUC participants encountered two of these (the HA and doctor). We first describe participants' experiences with specific human resources and then overall levels of satisfaction with the quality of care.

### Role of the lay health counselor (HC)

The HC was the key personnel in the CSC arm in both phases of MANAS trial and worked in collaboration with the primary care physician. Belonging to the local community, she delivered a range of psychosocial treatments including psychoeducation, interpersonal therapy, adherence management, referral to various agencies for social difficulties and offering yoga sessions where practical. More than three-fourth of the CSC participants in the PHC phase (*n* = 25; 81%) and half of the participants in the GP phase (*n* = 19; 50%) described the interactions with the HCs as beneficial. These participants depicted the HC as a good listener, problem solver, empathetic, caring, gentle, and supportive. Participants appreciated the HC's approach of helping the participant to understand his/her illness, probing about the psychosocial context and participants' beliefs and priorities, and suggesting various techniques for coping. These participants reported that communication with the HC was also essential to creating a good interpersonal relationship, information exchange and optimal decision making.

“She asked what is happening with me. I understood all her questions. She has to ask such questions about my health. The questions she asked were good. I told her whatever she asked. She patiently listened to my answers. She explained to me about the illness and why this is happening to me. She also gave me some handouts to read. I felt good. She told me not to take tension and follow the breathing exercise. I follow it up regularly and it has helped me. If I do it then my hands and legs become relaxed. I feel good. I like to talk with her.” (Male, 55, CSC)

Two third of participants from PHC phase (*n* = 21; 69%) and one fourth participants from the private phase (*n* = 8; 21%) cited the psychosocial interventions offered by the HC and breathing techniques, and yoga classes as having helped them in feeling relaxed, calming the mind, reducing psychological distress, keeping unwanted thoughts away and concentrating on daily activities.

“ …the (counselor) I met there, was good to me. She gave me some useful information and also told about the yoga and breathing exercises. This kind of information is not provided in other clinics. In those clinics, one just visits the doctor and comes back. But this was very helpful for me. These exercises helped in calming down and think in right direction. I feel much better now. I do not feel weak and fatigued. I feel like working. I now carry most of the household work. I feel this change is there because I follow the techniques suggested by the lady in the PHC (HC). She is good natured. Every time I meet her, she reminded me to take medicines on time.” (Female, 32, CSC)

CSC arm participants, mainly from the PHC phase, also reported that the HC was instrumental in helping them improving the disturbed relationship with the family members and significant others (such as neighbors). Two third participants from PHC phase (*n* = 20; 66%) and a smaller proportion from the GP phase (*n* = 5; 22%) reported that with the help of the HC, they could identify the problems in interactions with family members and/or with significant others and address them with suggested coping strategies. Some participants specifically cited that practicing yoga and breathing techniques has helped in decreasing anger and irritability toward other family members.

“…now I share a good relation with my daughter. I scold her sometimes when she does not inform me of coming late to home. Since I am in good health, I do not easily get angry. Now I also have a good relation with my sister-in-law. When I was not keeping in good health, I would easily get angry. Now I have a good relation with her. This change has occurred mainly due to the suggestions given by the counselor. She told me to use simple technique like counting backward when I feel angry and it is working for me”. (Female, 55, CSC)

CSC participants from both the phases appreciated the efforts taken by HC to reach out by sending letters or by making home visits. No participant from the CSC arm reported dissatisfaction with the counseling element.

### Role of the Health Assistant (HA)

The process of the screening interview with the HA was reported as a “therapeutic”, “good” or “helpful” experience by 68.5% (*n* = 37) of the PHC phase participants (*n* = 21; 68% in CSC and *n* = 16; 70% in EUC arm) and by 58% (*n* = 35) of the GP phase participants (*n* = 20; 52% in CSC and *n* = 15; 65% in EUC arm). They found this process helpful as someone listened to them carefully and this offered an opportunity for catharsis of their stressors. Thus, some HAs seemed to exceed their prescribed role of screening by also offering advice and exploring the participant's perspective (ideas, concerns, expectations, impact of condition on everyday life, etc.) about their illness and these were instrumental in relieving distress.

“I think this programme is good. Especially the girl (HA) who asked me some questions also helped me a lot. She asked me questions about everything. I felt really good. I thought someone is willing to talk to me. She also asked me to take my medicines on time.” (Female, 46, EUC)

However, about 11% (*n* = 5) EUC arm participants from both the phases reported that they were annoyed by questions asked the HA and expected more practical advice from her.

“…yes, I had to go to that girl [HA]. She asked me some questions, which I answered. When she asked me those questions I felt that somebody is trying to understand my problem. When somebody asks such questions, then one expects that the person would give some advice. I thought that after telling her about my problem, she would guide me to do something about my problem. But she did not”. (Male, 52, EUC)

### Doctor-patient interaction

In the PHC phase, marginally more participants from CSC than EUC arm reported satisfaction with the treatment received from doctors (*n* = 11; 35% vs. *n* = 6; 26%). The major determinant of the experience of the doctor–patient relationship was the extent and form of communication and exchange of information. The commonest complaint of the participants from both arms in the PHC phase was that doctors do not listen to them. Participants expected more and better information about their problem and the outcome, relief of pain and emotional distress, and advice on what they could do for themselves. Implying a paternalistic and distant relationship, participants said that the doctors would mostly limit the advice to taking medication regularly, not to worry and not to get tensed without elaboration of what tension and worries entail and the different ways of coping with it.

“…I told my doctor to check my pressure, he refused to check. They (PHC doctor) do not check properly. The treatment is very poor…doctors in the PHC treat patients like animals. They need to ask the patient what is happening, examine the patient…should show concern to the patient. They do it only for the sake of doing; they would say that pressure is normal and then write the prescription and send the patients.” (Male, 46, EUC)

Five participants from CSC and two participants from EUC arm reported the doctor as being concerned, empathetic and kind but constrained because of large number of participants at PHC and limitation of time.

In the GP phase, between a third and half of participants (*n* = 20; 53% from CSC; *n* = 8; 34% from EUC arm) found the experience with and treatment of the doctor as positive. These participants reported satisfaction with *how* doctors communicate, for example how they discover the history or provide information, and the verbal and non-verbal skills they use. They also reported satisfaction with *what* the doctors explained about the nature of the illness, treatment management and adherence. These participants also described how they develop the relationship with the doctor, how they have been going to the particular doctor for a number of years and how s/he knows everything about their family health problems.

Some of the participants attributed their improvement to the physician's awareness of their life circumstances and problems, willingness to experiment with different medications, and treating patients with dignity. It appears that for these participants the faith in their physician was crucial for bringing positive change.

“Doctor XXX's words and touch itself is enough to heal the patient. He is a good doctor and talks affectionately with us. He also cracks some jokes, he has that habit of making everything light and when you get such a doctor you feel like going to the same doctor. I experienced a change in myself after visiting Dr. XXX. I felt that there is someone to listen to you and at least someone is concerned about you if not your family members and this is there in the clinic.” (Female, 46, EUC)

### Satisfaction with treatment and care

In the PHC phase, more than three fourth of CSC participants (*n* = 25; 80%) reported satisfaction with the overall treatment and care received; however most of the participants could not describe the components of treatment that they found to be the most helpful or were satisfied with. In comparison, fewer participants from EUC arm (*n* = 10; 44%) reported satisfaction with the overall treatment and care received. A greater number of EUC participants made decisions regarding treatment discontinuation (*n* = 10; 44% vs. *n* = 3; 10%) or seeking help from other healthcare providers (*n* = 11; 48% vs. *n* = 4; 13%). Around half of CSC participants could not differentiate between the HA and HC and found the interaction with and information shared by them as “useful”. More than half participants from both the arms described that the addition of the HA and/or HC being “helpful” and “something new” and the treatment received in PHC has made a difference in their life.

The most commonly mentioned barriers by participants from both arms to accessing and maintaining treatment were financial reasons i.e. the cost of the transport to the PHC and the added cost of buying medications that were not included in the program (*n* = 22; 41%). Other barriers included: transient recovery from symptoms and thus stopping treatment without further consultation; day-to-day life concerns which would come in the way of attending the clinics; a long waiting time at the PHC; and dissatisfaction with the care received at the PHC.

“…doctors at the PHC are very “careless”…gave me tablets for vomiting and injection which I had to buy from private pharmacy…I require money for transport, doctors could have done something; they need not admit me in the hospital but could have put at least glucose for strength. Why should I go to the PHC? At PHC doctors only prescribe medicines. If it is a lady doctor, she does not even examine the patient. PHC doctors do not care” (Female, 50, CSC)

During the GP phase, nearly two-thirds of CSC participants (*n* = 23; 61%) compared to less than half of the EUC participants (*n* = 9; 40%) reported overall satisfaction with the care and described it as being “helpful”; as with the PHC participants, most could not describe whether any specific component of care was found to be helpful. Nearly two-thirds of CSC participants (*n* = 23; 61%) and half of the EUC participants (*n* = 12; 53%) reported the atmosphere in the clinic as “supportive”. More participants from the EUC arm than the CSC arm discontinued the treatment from the GP as they felt it was not benefiting them (*n* = 10; 44% vs. *n* = 10; 28%). The most common reasons for discontinuation in both arms were financial constraints (*n* = 5, 13% in CSC; *n* = 8, 35% EUC) and lack of relief from the symptoms or side effects of the medication such as drowsiness and heavy headedness (*n* = 5, 13% in CSC; *n* = 2 8% in EUC). Half the CSC participants (*n* = 12; 53%) thought that information shared by the HC was “useful” and helped in making better decisions. However, nearly one fourth of participants from both the arms (*n* = 9 24% in CSC; *n* = 5 22% in EUC) did not understand the purpose of screening and thought that it was waste of time.

### Impact on health

Prior to commencing treatment, participants in the intervention and control arms used the psychological construct of “tension” and “worry” to describe their illness and complained of a significant impairment in their lives due to the prevalence of somatic complaints (especially pain symptoms, disturbed sleep, palpitation, giddiness, numbness, gastrointestinal symptoms and reproductive health symptoms) and emotional complaints (weakness, lack of energy, inability to concentrate, memory problems, sadness, social isolations, suicidal ideation and rumination of thoughts) (Andrew, Cohen, Salgaonkar, & Patel, 2012).

#### PHC (Public facility) phase

Post intervention, more than three-fourths of the participants (*n* = 26; 83%) from CSC arm reported relief from a range of symptoms, using terms like “good” and “better”. CSC arm participants reported striking benefits in sleep and somatic complaints like pain and aches. Many participants linked improvements in somatic symptoms with the improvement of emotional symptoms.

“Now, I am in good health; I could concentrate on my studies. I am not worried about my relationship with my mother… Now I am better, I don't feel weak and tired, I don't get those thoughts, I don't feel fed up. I also don't get palpitations. Even during the exams I was better. I was eating well. My sleep is always good. I would get thoughts of what she (mother) would say to me, I tried to divert my thoughts by concentration on my studies and other activity like watering the garden. The medicines (ADT) prescribed by the doctor and the techniques suggested by the lady (counselor) has helped me a lot.” (Female, 20, CSC)

The majority of participants (*n* = 23; >90%) from CSC arm who reported improvement attributed this to the medicines and the HC intervention.

In the EUC arm, fewer participants reported improvement (*n* = 13; 55%) and the improvement was limited to the somatic complaints. At the follow-up interview, four participants also reported that this relief in symptoms was temporary and they had experienced a relapse. More than half the participants (*n* = 9; 69%) attributed relief from symptoms to the medicines prescribed by the doctors and four attributed it to the interaction with the HA (31%).

“At present, all my health complaints are the same. I do not get good sleep; my body ache has become worse, I feel I have no appetite; I feel full. I visited the PHC twice, first time I was given tablets (ADT), I felt little better with it. Then in my second visit, I was given tablets (Antacid, Rantac and Calcium). Actually, I used to visit a private doctor but could not meet him lately as he has gone to Mumbai. In between, I had taken my daughter to a private doctor nearby as she was not keeping well. I too showed myself to the doctor for my problem. Now my pain has reduced. There is improvement.” (Female, 52, EUC)

No participant from the CSC and EUC arm felt that the intervention has caused any harm.

#### GP (Private facility) phase

The majority of participants from the CSC arm (*n* = 31; 82%) reported relief. Participants described recovery as a gradual process during which they experienced relief from somatic symptoms, e.g., aches, pain and sleep problems, as well as psychological symptoms, e.g., sadness, emptiness, irritability, and hopelessness. Three-fourths (*n* = 23; 75%) of the CSC arm participants who experienced improvement attributed this change to the intervention, in particular the medicines prescribed by the GP; about half (*n* = 16; 52%) recognized the HC as a contributory factor in recovery.

“My health has improved; after taking the tablets (ADT) my health has been good. I do not get thoughts. I don't feel worried. I feel like working in the household. I do not worry about my family. The medicines given to me are very effective. I also practise the techniques like stretching suggested by the lady [HC] in the PHC and I feel better.” (Female, 55, CSC)

In the EUC arm, two third participants (*n* = 15; 67%) reported improvement and attributed it to the medication prescribed by the physician. Most of these participants reported of experiencing change in their daily life with improved sleep and eating pattern, gain in energy and strength, and reduction in worries and sadness.

“I feel much better now; I feel my health is improving, after taking the medicines (ADT) prescribed by the doctor, there is an improvement in my health. I do not feel sad or tensed. In fact, I have started doing the household work.” (Female, 32, EUC)

No participants from CSC and EUC arm reported any harm due to the intervention.

### Impact on social functioning

Prior to the intervention, approximately two-thirds of participants from both phases reported that depressive symptoms caused strain in their relationships with family members and significant others (Andrew et al., 2012). They described ‘going into hiding’, canceling or postponing work, avoiding friends, or having family troubles. About two-thirds of participants expressed a causal link between illness and family relationships, and reported worsening of interactions due to anger and irritation with other family members. More than three-fourths of participants from both phases also described that the illness interfered with their ability to work effectively within the household and workplace. Lack of energy and fatigue were common experiences and participants often described this phenomenon as *‘bejar ailo’* (feeling fed up). Some participants described that not being able to work increased their “worries” and “tensions” and had an impact on their earnings and livelihood, which in turn had an adverse impact on their relationship with family members.

#### PHC (Public facility) Phase

More than two-thirds of CSC arm participants (*n* = 25; 81%) reported that the intervention had a positive impact on their relationship with other family members. Participants whose families were experiencing financial difficulties described an improvement in their attitude by “letting go”, accepting things as they were, being constructive in problem-solving and trying to not think about them too much.

“…my attitude has changed. I talk very quietly and nicely with my husband, children and daughter-in-law. I tell my husband not to worry and motivate him to keep searching for a job. I reassure him that I am feeling better with these tablets and will continue to feel the same by making regular visits to the PHC. I try to understand his situation by not arguing or shouting at him when he yells or shouts. I need to think about his health too. Even if we are having financial problems, we will manage what we have”. (Female, 50, CSC)

Most CSC arm participants (*n* = 27; 87%) reported improved energy level and abilities to carry on their daily routine and work after receiving the intervention, with improved efficiency and concentration. Participants attributed this to relief from symptoms such as tiredness, weakness and lack of interest, and energy as a result of the medication and advice given by the HC.

“…now I enjoy doing household work, I don't feel fed up since I am in good health. I can do all the household work, from washing, cooking, cleaning to fetching water…after taking all the tablets (ADT) prescribed by the PHC doctor I felt better. I do not feel weak and enjoy doing household work. Now my health is better and I can do all my work. I also do yoga and breathing exercises which make me feel relax.” (Female, 50, CSC)

However, four CSC participants reported that in spite of improved symptoms, they were unable to work as before; when they tried to work, they experienced aches, pains, heaviness in the chest and head, giddiness and palpitations.

Only half of the EUC participants (*n* = 12; 52%) reported improvements in their relationship with family members. These participants also attributed the improvements in relationships to the improved mood and control over anger and irritation. Around one-third of the participants (*n* = 7; 30%) reported improvement in their energy levels and ability to work and attributed this positive change to the medication received, a change in interpersonal relations, improvement in socio-economic environment (e.g., finding a job, improvement in the economic condition, etc.) and benefits of health care, including that received from other private healthcare providers.

“ …when I was at home; I was feeling bored. I felt tense but that is a past now. In these two months, treatment has helped me (ADT). Initially, when I started working, I had no one to talk to but then I got used to it. I had good concentration while I was working. I cooked my own food. I did it with a lot of enthusiasm. It was not an easy job but I enjoyed doing it. I did not get bored. This season (monsoon season), I will work in my fields too.” (Male, 64, EUC)

More than one-third of EUC participants (*n* = 14; 38%) reported that in spite of improved symptoms, they were unable to work, and they experienced fatigue, aches, pains, giddiness and palpitations.

#### GP (Private facility) phase

A minority of participants from the CSC arm (*n* = 7; 18%) reported a change in their relationships and mostly attributed this to the advice received from their physician and changes in their economic conditions. Five participants (13%) reported that their relationships, either with family members and/or significant others which were strained before the onset of the illness had remained unchanged despite the intervention. Half of the CSC participants (*n* = 19; 50%) reported being able to carry out their daily work effectively as a result of improved energy levels and concentration, and relief from somatic and emotional symptoms. Five participants (13%) attributed this positive change to the advice received from the HC.

“There is a change in my behavior. Now I don't get angry on my children and beat them. I don't argue with my husband. I feel that it is because I met the counselor in the doctor's clinic.” (Female, 40, CSC)

However, six CSC participants (16%), mainly women, expressed that they felt like working but were unable to do so due to aches and pains.

“…I like to work but I can't as I do not have strength. I get aches and pain if resumed working. I feel giddy; I feel that I will fall down. Earlier I was unable to prepare breakfast for my children but now I can do only that much.” (Female, 40, CSC)

Five EUC participants (22%) reported a positive change in their relationships of which one attributed it to the treatment received from the GP while remaining linked it with the change in their social environment and/or treatment received from an *ayurvedic* doctor.

“Earlier I would get annoyed with my children and husband but now it is changed. I am feeling better. I think that it is only because of the ayurvedic medicine.” (Female, 35, EUC)

Two participants who had reported that their relationship with a family member was estranged due to the illness expressed that the treatment from the GP had produced no impact.

The majority of EUC participants (*n* = 20; 87%) reported no change in their ability to work; those who benefited attributed this to change in their social circumstances, medications received from doctors or traditional healers, and prayers.

## Discussion

This paper reports the findings of a qualitative investigation of the impact and experience of a lay health counselor led collaborative stepped care intervention, compared with enhanced usual care, for common mental disorders (CMD) in public and private primary care in Goa, India. Patients in both arms encountered a new health personnel (the Health Assistant) in the clinic who carried out the screening to detect CMD in addition to their doctor. In the intervention arm, an additional personnel was the Health Counselor (HC), the lay health worker who provided case management and psychosocial interventions. Our primary findings are that when compared with enhanced usual care, more participants in the intervention arm reported relief from symptoms, and an improvement in social functioning in terms of relationships with other family members and significant others and positive impact on work and activities in day-to-day life. The intervention arm participants attributed their improvement to both medication received from the doctors and the strategies suggested by the HCs. Greater number of participants from the intervention arm also reported satisfaction with the overall quality of the care. Contact with the HA was also found to be beneficial by participants in both arms. Participants from control arm were more likely to seek multiple treatment sources and engage in ‘doctor shopping’ due to lesser satisfaction with provision of services and perceived impact of care received. However, some key differences were observed in the results for the private and public facilities.

The quantitative evaluation revealed that the intervention was consistently associated with strong beneficial effects over the 12 months on impact on mental health and disability outcomes in public facilities (Patel, et al., 2011). The results obtained through this qualitative investigation confirm that a collaborative-stepped care intervention delivered by HCs can improve recovery rates for patients with CMDs in public PHC settings. Though two key components of the intervention (provision of screening results to participants and physicians, and evidence-based guidelines to the physician) were offered in both the groups, the qualitative investigation indicate that the differences in the results are due to the instrumental role played by HCs in intervention arm. The intervention arm participants considered the HCs to be an important component of providing care who served as a link between patient and the doctor, provided them skills on stress management and helped in adherence to medication. In the PHCs, larger numbers of patients are seen by the doctor in a shorter period and hence the setting and time constraints limit the ability of the doctor to discuss the interpersonal and social difficulties and issues related to treatment management and adherence. Our findings indicate that this gap was filled by the HCs and to some extent by HAs, with whom participants could discuss range of issues related to illness as well as interpersonal and social difficulties. It is well established that a positive therapeutic relationship in which patients feel free to discuss their problems and work toward their resolution is related to improved outcomes from treatment, particularly in primary care (Anderson, Lindberg, & Troein, 2002; Cape, 2000). These findings are also consistent with those obtained from semi-structured interviews with healthcare providers of the MANAS trial (Pereira et al., 2011). The PHC healthcare providers recognized the importance of screening and the categorization of the severity of CMD by the HA and reporting of the results on a patient card as an aid in diagnosis and providing treatment, which helped them in overcoming the challenges of the shortage of time and the common presentation of somatic symptoms. These health care providers also valued the contribution of HCs who provided a range of psychosocial treatments and emphasized adherence management which widened the scope of health care interventions and enhanced the likelihood of success.

Quantitative findings of the MANAS trial also revealed significant effect modification by type of site, i.e. the recovery rates were not significantly different between the two arms in the private facility phase because the control arm participants had similar recovery rates as those of the intervention in either phase (Patel et al., 2011). In part, our qualitative findings confirm that the greater effectiveness of the private control arm (compared with the control arm of the public facilities) was due to the nature of the therapeutic relationship between patients and doctors. Thus, participants described sharing a long-term and trusting relationship with the doctor. Participants from both arms placed faith in the doctor who also acted as a confidante, was known to the patient for a long period of time, and was perceived to understand the patient's health and context intimately. Unsurprisingly, private sector participants were more likely to report satisfaction with the communication and treatment provided by the doctor. In addition, majority of the beneficiaries, even from the intervention arm, associated improvement to the antidepressants rather than to the HC component of intervention. Thus, it seemed that to a large extent the treatment and care provided by the private doctors approximated the care provided by the HCs. In addition, some participants also reported that interaction with HAs was helpful and therapeutic, and served as a catharsis of their stressors in life; indeed, some participants in the intervention arm confused the roles of the HC with the person carrying out the screening. Although the qualitative investigation observed that greater numbers of participants from the intervention arm than control reported relief from the symptoms, there were fewer differences in improvement in interpersonal relations, work life and perceptions of quality of care. These findings are consistent with those obtained from semi-structured interviews with private GPs of the MANAS trial (Pereira et al., 2011). The interviews with the control arm GPs revealed a number of practices which approximated the program interventions to the extent to which these might well explain their comparable performance. Thus, most GPs routinely diagnosed CMD, and provided psychoeducation, advised on life style changes and problem solving. These GPs also routinely prescribed ADT and referred patients with severe CMD to a psychiatrist. Above all, GPs developed good rapport with patients, offering one-to-one consultations in a private space, maintaining confidentiality, and offering advice which reflected their long-standing relationship with the patient and understanding of the patient's social context. Hence, it appears that there is little advantage in adding a new human resource in the form of a counselor/case manager in private primary care settings, though the addition of screening may be of some additional value.

Although there are now several trials conducted on collaborative stepped care approach to manage depression in the primary care settings in the high and middle income countries, to the best of our knowledge, MANAS is the first trial evaluating the impact of the intervention through concurrent quantitative and qualitative investigation. In conclusion, results from this qualitative investigation indicate the effectiveness of a lay health counselor-led collaborative stepped-care intervention for CMDs in public PHCs on a range of outcomes including symptomatic relief, social functioning and satisfaction with care. The intervention used in the current study may provide a model of how a trained lay counselor can assist the busy public sector primary care physician in educating patients, monitoring and reinforcing adherence, and helping patients with behavioral and lifestyle changes that may be beneficial to their clinical outcome. In the private sector, training GP's in detection, treatment and care of common mental disorders may be sufficient to achieve comparable outcomes. Policy makers concerned with public health care should consider including lay health workers as a key human resource for delivering collaborative care for CMDs in resource constrained, busy public primary care facilities.

## Figures and Tables

**Table 1:** Characteristics of participants in MANAS trial qualitative study.

Characteristics	PHC phase			GP phase		
	CSC arm	EUC arm	Total	CSC arm	EUC arm	Total
Completed 1st interview	31	23	54	38	23	61
Completed 2nd interview (%)	25 (80.6)	19 (82.6)	44 (81.5)	30 (79)	19 (82.6)	49 (80.3)
Mean age (years) (SD)	51 (11.01)	45 (13.23)	47.6 (12.87)	50.7 (15.81)	45.7 (12.16)	48.8 (14.61)
Gender (%)						
Male	12.9	26.0	18.5	36.8	26.0	32.8
Female	87.1	74.0	81.5	72.2	74.0	61.2
Mean GHQ score (SD)	8.8 (1.13)	9.34 (1.02)	9.05 (1.16)	8.57 (0.72)	8.65 (0.69)	8.60 (0.71)

## Box 1 An overview of the MANAS trial

**Table:**

Aim: To evaluate the effectiveness and cost-effectiveness of a collaborative stepped care intervention led by lay health counselors in public and private primary care facilities to improve the outcomes of people with common mental disorders (CMD)
Study setting and duration: Goa India; 12 private general practitioners and 12 public primary health care facilities (April 2007 to September 2009)
Design: Cluster randomised controlled trial with health facility as a unit of randomization; randomization stratified by type of facility (private or public)
Inclusion criteria: Participants who were 18 years old and above; had a Common Mental Disorder i.e. a score of 6 or above on the General Health Questionnaire-12 (GHQ); resident in Goa for the subsequent 12 months; speaking Konkani, Marathi, Hindi or English; and consented to a home visit for outcome assessment. Exclusion criteria: Participants requiring urgent medical attention; and having difficulty with hearing, speaking or cognition that makes interviewing difficult
Total N: 2796 participants (1648 in public sector, and 1148 in private sector)
Intervention: The Collaborative Stepped Care (CSC) multi-component intervention was developed through a systematic process based on the MRC framework for the development of complex health care interventions (Craig et al., 2008). The findings of this formative work and the final intervention are published elsewhere (Chatterjee et al., 2008). The intervention comprised the following components: case management, psychoeducation, interpersonal psychotherapy, antidepressants (for moderate/severe CMD), adherence support and referral to social welfare agencies. In addition, yoga was offered as an adjunctive intervention in those clinics where space was available. All psychosocial components were delivered by a lay health counselor (HC) and medication by the primary care physician. A mental health specialist provided support and supervision to the PHC team. The intervention components were delivered in a four step fashion (step 1: psychoeducation; step 2: antidepressant or psychotherapy; step 3: antidepressant plus psychotherapy; step 4: referral to a psychiatrist) with clear rules on progression between steps.
Comparison arm: Enhanced Usual Care (EUC) comprised providing the primary care physicians with the results of the diagnostic screening and guidelines for the treatment of CMD. There were no additional human resources.
